# Surgical Stabilization of the Spine: A Clinical Review of Spinal Fractures, Spondylolisthesis, and Instrumentation Methods

**DOI:** 10.3390/jcm14041124

**Published:** 2025-02-10

**Authors:** Adrian-Valentin Enache, Corneliu Toader, Razvan Onciul, Horia Petre Costin, Luca-Andrei Glavan, Razvan-Adrian Covache-Busuioc, Antonio-Daniel Corlatescu, Alexandru Vlad Ciurea

**Affiliations:** 1 Sanador Clinical Hospital, 010991 Bucharest, Romania; adrian-valentin.enache@drd.umfcd.ro (A.-V.E.); prof.avciurea@gmail.com (A.V.C.); 2 Department of Neurosurgery, Carol Davila University of Medicine and Pharmacy, 050474 Bucharest, Romania; razvan.onciul@drd.umfcd.ro (R.O.); horia-petre.costin0720@stud.umfcd.ro (H.P.C.); luca-andrei.glavan0720@stud.umfcd.ro (L.-A.G.); razvan-adrian.covache-busuioc0720@stud.umfcd.ro (R.-A.C.-B.); 3 National Institute of Neurology and Neurovascular Diseases, 050474 Bucharest, Romania; 4 Department of Orthopedics and Traumatology, Carol Davila University of Medicine and Pharmacy, 050474 Bucharest, Romania; antonio.corlatescu@gmail.com

**Keywords:** spinal fractures, spondylolisthesis, spinal stabilization, minimally invasive surgery, vertebral augmentation

## Abstract

The spine is a complex structure critical for stability, force transmission, and neural protection, with spinal fractures and spondylolisthesis posing significant challenges to its integrity and function. Spinal fractures arise from trauma, degenerative conditions, or osteoporosis, often affecting transitional zones like the thoracolumbar junction. Spondylolisthesis results from structural defects or degenerative changes, leading to vertebral displacement and potential neurological symptoms. Diagnostic and classification systems, such as AO Spine and TLICS, aid in evaluating instability and guiding treatment strategies. Advances in surgical techniques, including minimally invasive approaches, pedicle screws, interbody cages, and robotic-assisted systems, have improved precision and recovery while reducing morbidity. Vertebral augmentation techniques like vertebroplasty and kyphoplasty offer minimally invasive options for osteoporotic fractures. Despite these innovations, postoperative outcomes vary, with challenges such as persistent pain and hardware complications necessitating tailored interventions. Future directions emphasize predictive analytics and enhanced recovery strategies to optimize surgical outcomes and patient quality of life.

## 1. Introduction

The spine is a complex, multi-joint structure supported by muscular control, which enables head and trunk stability across different postures and movements. It also serves as a crucial protector of the spinal cord, nerve roots, and the vertebral arteries in the cervical region. Proper spinal function depends on stability, which not only shields neural structures but is vital for efficient force transfer between the upper and lower body, generating forces in the trunk, reducing biomechanical wear on spinal components, and conserving muscular energy [1,2].

Although various biomechanical and clinical perspectives have defined spinal stability, a universally accepted definition still needs to be discovered. Spinal instability, or compromised stability, is a significant yet often overlooked factor contributing to back pain, particularly in the lumbar region. Advances in mobile stabilization systems aim to neutralize harmful forces, restore normal spinal segment function, and protect adjacent structures, marking a new frontier in treating degenerative spinal conditions. These systems are receiving increasing attention from bioengineers and surgeons. According to the American Academy of Orthopedic Surgeons, stability is “the capacity of the vertebrae to remain cohesive and to preserve the normal displacements in all physiological body movements” [3].

Advancements in vertebral and pedicle screw fixation have improved spinal stability while reducing long-term immobilization. King first described lumbosacral screw placement in 1948. Minimally invasive spine surgery evolved alongside laparoscopic techniques, starting with chemonucleolysis in 1964 and microdiscectomy in 1977. The TLIF technique, introduced by Harms and Jeszenszky in 1998, was refined into a minimally invasive approach by Foley in 2003. Robotics further revolutionized spine surgery, with FDA-approved systems like SpineAssist (2004), ExcelsiusGPS, and ROSA Spine, enhancing accuracy and reducing radiation exposure [4].

Each vertebra within a spinal motion segment (MS), or functional spinal unit (FSU), performs various movements, with stability maintained by an array of bony and soft tissue structures. This intrinsic complexity, however, complicates clinical and imaging assessments of spinal movement. Similarly, spinal instability is yet to have a broadly accepted definition. Determining “normal or physiological movements” is challenging, as significant overlap in movement types exists between symptomatic and asymptomatic individuals. This complicates the establishment of standard diagnostic references and the correlation of clinical symptoms with imaging findings [5].

Spinal fractures pose a significant threat to the stability of the spinal column, a complex and dynamic system designed to remain stable under normal physiological conditions. The primary objective in spine trauma surgery is to restore this stability and structural integrity after injury. Demographic shifts, particularly an aging population and advancements in accident prevention, have influenced both the frequency and nature of spinal trauma, necessitating changes in treatment approaches. Innovations in spinal instrumentation and an improved understanding of spinal biomechanics and sagittal balance have further enhanced surgical strategies for treating spinal fractures. Fractures are most commonly observed at the thoracolumbar junction, where the relatively rigid thoracic spine transitions to the more flexible lumbar region, resulting in increased biomechanical stress. Additionally, about 17% of spinal fractures occur at the cervicothoracic junction, highlighting the susceptibility of these transitional zones to traumatic forces [6].

In 2017, across the five most populous countries in the European Union, along with Sweden (EU6), an estimated 2.7 million fragility fractures occurred, resulting in an annual cost of EUR 37.5 billion and a loss of one million quality-adjusted life years (QALYs). By 2030, these numbers are projected to increase by 23%, translating to 3.3 million fragility fractures and a 27% rise in associated healthcare spending (1). In Spain, the healthcare cost of osteoporotic fractures is substantial, representing 3.8% of total healthcare expenditure in 2019, equivalent to EUR 4.3 billion in direct costs, excluding the loss in QALYs—a proportion slightly above the European Union average of 3.5% [7].

Regarding health-related quality of life (HRQoL) after fractures, a population-based study conducted in Denmark involving 12,839 men and women over 50 years of age with fractures and 91,246 controls revealed significant deficits in both physical and mental components of the 12-item Short Form Health Survey (SF-12) among fractured patients. Hip and vertebral fractures were found to have the greatest impact on HRQoL, with deficits persisting up to five years post-fracture. Additionally, when assessing the specific impact of socioeconomic status (SES) on HRQoL in fracture patients, those with low SES demonstrated greater deficits in the mental component of the SF-12 compared to individuals with high SES, though no difference was observed in the physical component [8].

Assessing spinal stability is critical in managing spinal fractures, as compromised stability can lead to significant instability, impacting overall spinal function. Effective evaluation of these injuries includes a comprehensive clinical history, detailed physical examination, and structured radiographic assessment to determine the injury’s mechanical stability, neurological involvement, and specific patient factors that may influence recovery. According to White and Panjabi, spinal stability is defined as the spine’s capacity, under physiological loads, to limit displacement to prevent damage to the spinal cord or nerve roots, thereby avoiding severe deformity or pain due to structural changes [9]. When spinal instability is identified, surgical or nonsurgical treatment aims to restore stability, reduce neurological damage risks, and prevent long-term structural complications.

Adjacent segment disease (ASD) is a common concern following lumbar fusion surgery, encompassing both radiographic changes (ASDeg) and clinical symptoms (ASDis). While minimally invasive surgery (MIS) and traditional open approaches differ in surgical techniques and impacts on surrounding structures, long-term studies, such as Jeong et al. [10], have demonstrated comparable ASD rates between the two techniques after a 10-year follow-up, with incidences of 34.9% and 31.8%, respectively. Despite these similarities, the MIS approach reduces facet joint violation and soft tissue damage, potentially preserving adjacent segments more effectively. Beyond surgical outcomes, the psychological dimension of chronic postoperative pain warrants greater focus, as prolonged discomfort can lead to depression and anxiety. Integrating psychological support into a multidisciplinary treatment framework can significantly improve patient outcomes, fostering better pain management and quality of life. The inclusion of mental health professionals in postoperative care may reduce the psychosocial burden and optimize recovery trajectories.

Spondylolysis is a structural defect in the back portion of a vertebra, specifically in the pars interarticularis [11]. This condition typically arises due to trauma or the cumulative effects of repetitive loading and excessive extension of the spine. When this instability allows the vertebral body to shift, it results in a condition known as spondylolisthesis [11,12]. This shift generally requires either a fracture or deformation in the posterior spinal elements, which causes the pars interarticularis to elongate. Spondylolysis can affect individuals of any age, although the causes may differ. If the vertebral displacement progresses to cause neurological symptoms, surgery may be needed to relieve pressure and stabilize the vertebrae [13]. However, if no motor deficits are present, the preferred initial treatment involves conservative measures, including pain management, activity modification, and possibly therapeutic injections over several months [14].

## 2. Spinal Fractures

Vertebral fractures constitute a significant clinical entity, with etiology spanning from high-energy traumatic injuries to low-energy osteoporotic fractures. While osteoporosis remains a predominant risk factor for vertebral fractures in the elderly, it is imperative to acknowledge the bimodal distribution of vertebral fractures across different patient populations. High-energy traumatic fractures predominantly affect younger individuals and are commonly associated with falls from significant heights, motor vehicle collisions, and high-impact sports injuries. Conversely, osteoporotic fractures primarily occur in elderly individuals following low-impact events, such as falls from standing height, due to compromised bone quality and reduced mineral density [15].

Recent epidemiological analyses emphasize that traumatic vertebral fractures in younger individuals often necessitate immediate surgical intervention to restore spinal stability, whereas osteoporotic fractures may be managed with conservative treatment, vertebral augmentation, or minimally invasive procedures. A population-based study further highlights that vertebral fractures in the elderly are associated with significant long-term morbidity, reduced quality of life, and an increased risk of subsequent fractures, underscoring the importance of early diagnosis and fracture prevention strategies [15,16].

Osteoporosis affects over 200 million people globally [17], with a prevalence of up to 30% among women [18]. In the United States, vertebral compression fractures impact more than 1.5 million individuals annually, with incidence rates of 10.7 per 1000 women and 5.7 per 1000 men [19,20].

Osteoporosis plays a critical role in the pathophysiology and prevention of spinal fractures, particularly in the elderly population, where it is a leading contributor to vertebral compression fractures (VCFs). The structural weakness caused by osteoporosis results from a combination of decreased bone mineral density, compromised bone quality, and disrupted microarchitecture. These changes significantly increase the risk of fractures, often triggered by minimal trauma, such as a fall from standing height. VCFs not only lead to acute pain and spinal deformities but also initiate a vicious cycle of immobility, comorbidities, and heightened mortality risk. According to Komadina et al. [21], timely identification and management of osteoporosis are essential for reducing fracture incidence and improving long-term outcomes. Dual-energy X-ray absorptiometry (DXA) and advanced imaging techniques, such as MRI, are invaluable for diagnosing osteoporosis and assessing fracture severity. Incorporating pharmacological treatments, such as bisphosphonates or denosumab, alongside minimally invasive techniques like vertebroplasty and balloon kyphoplasty, can mitigate the progression of fractures and enhance the quality of life in affected individuals.

Recent evidence suggests that percutaneous vertebroplasty (PVP) is a safe and effective treatment option for osteoporotic burst fractures, even in cases with asymptomatic spinal canal compromise. A five-year follow-up study demonstrated significant improvements in pain relief and functional outcomes, as measured by the visual analog scale (VAS) and Oswestry Disability Index (ODI), with no notable differences in adjacent fracture rates or long-term complications when compared to vertebral compression fractures. While traditional guidelines often discourage the use of PVP for burst fractures due to concerns over cement leakage and neurological risks, this study highlights that, with meticulous technique and patient selection, these risks can be mitigated. Additionally, the procedure was shown to effectively maintain vertebral height and reduce kyphosis without worsening retropulsed fragments, making it a viable option for managing select osteoporotic burst fractures [22].

Recent evidence underscores the efficiency and safety of repeated vertebroplasty (VP) in managing adjacent segment fractures (ASFs), a common complication following primary VP procedures. A recent study demonstrated that repeated VP significantly improves radiographic parameters, such as reducing kyphotic angles and restoring anterior and mid-vertebral body height, while also providing substantial pain relief and functional improvement, as evidenced by decreases in visual analog scale (VAS) and Oswestry Disability Index (ODI) scores. Despite these benefits, the procedure is associated with a notable complication rate, including cement leakage, de novo fractures, and occasional neurological deficits, emphasizing the importance of careful patient selection and surgical precision. These findings suggest that repeated VP remains a viable option for ASF treatment, particularly in elderly patients with comorbidities, where minimally invasive interventions are often preferable to open surgical alternatives [23].

A diagnosis of vertebral compression fracture significantly increases the risk of subsequent fractures, with a fivefold increase following one fracture and a twelvefold increase after two fractures [24,25]. These fractures are linked to both acute and chronic pain, diminished quality of life, decreased self-esteem, social isolation, higher fall risk, additional fractures, and a mortality rate nearly twice that of matched controls [26]. Osteoporotic compression fractures most frequently occur at the thoracolumbar junction, particularly between T11 and L2. Traumatic spinal fractures, with an estimated incidence of 160,000 cases annually, also commonly involve the thoracolumbar junction, accounting for approximately 50% of cases. These injuries are more frequent in men, with an average affected age of 30 years [27].

A vertebral compression fracture (VCF) is defined by a reduction in vertebral height of at least 15% to 20%. Although these fractures can occur at any spinal level, they are most often observed in the lower thoracic region. In individuals with advanced osteoporosis, VCFs may result from minimal activities, such as coughing or moving in and out of a bathtub. Compressive damage from VCFs typically affects the anterior portion of the vertebral column, making these fractures relatively stable and rarely associated with nerve root irritation or spinal cord injury [28].

Traumatic spine injury (TSI) is a significant contributor to spinal fractures. In the United States, the annual incidence of TSI is approximately 40 cases per million people, translating to about 12,000 new cases yearly [29]. The primary mechanisms of spinal cord injuries, ranked by frequency, are as follows [29]:

- Motor vehicle collisions (42%);
- Falls (27%);
- Violence-related acts (15%);
- Sports injuries (8%);
- Other causes (9%).

In more than half of the cases, spinal injuries occur in isolation [30], while approximately 25% of patients sustain concurrent injuries to the brain, chest, or major extremities [31]. Although TSI is traditionally associated with young males, recent epidemiological studies indicate a bimodal distribution [32]. The first peak in incidence is observed among adolescents and young adults, while a second peak is now evident in the elderly population (age > 65 years).

The AO Spine and TLICS (Table 1 and Table 2) classification systems are widely used in clinical practice to assess vertebral compression fractures (VCFs). Combining radiological findings with clinical factors like neurological deficits and spinal stability provides a comprehensive evaluation of fracture severity. Backed by extensive research, these systems are reliable tools for guiding treatment decisions.

The reliability of the AOSpine Thoracolumbar Injury Classification System (ATLICS) is essential for effective decision-making in clinical practice. Since 2013, a treatment algorithm based on Kepler et al.’s Thoracolumbar AOSpine Injury Score (TL AOSIS) [33] has been integrated into the system. This scoring system assigns points based on the severity within three subgroups: morphological, neurological, and case-specific modifiers. The total injury score serves as a guide for determining whether surgery should be considered and may also help predict the optimal timing for surgical intervention, as highlighted in the study by Du et al. [34].

Radiographs are often the first imaging technique used when vertebral compression fractures (VCFs) are suspected or found incidentally. They effectively assess overall spinal alignment, detect fractures, and identify signs of vertebral collapse or deformity [35]. VCFs frequently produce a wedge-shaped deformity in the vertebral body due to anterior collapse, resulting in a trapezoidal appearance on radiographs. The vertebral body may lose its normal contours and exhibit a burst appearance in severe cases.

CT imaging provides a detailed three-dimensional analysis of VCFs, making it invaluable for evaluating complex fractures [36]. Common CT findings include fracture lines, vertebral body collapse, cortical integrity loss, retropulsion, spinal canal stenosis, and the involvement of posterior elements. In acute fractures, CT often reveals the fracture line within the vertebral body, presenting as a disruption in the normal bony architecture. These fracture lines may appear linear or take on more complex patterns like wedge or burst fractures. CT imaging also allows precise measurement of vertebral body height loss by quantifying the anterior, middle, and posterior heights, and it helps assess cortical disruption associated with VCFs.

High-resolution magnetic resonance imaging (MRI) plays a pivotal role in the diagnostic evaluation of spinal pathologies, particularly in assessing ligamentous injuries, spinal cord conditions, and adjacent bony structures. MRI offers superior soft tissue contrast and detail compared to computed tomography (CT), making it indispensable for detecting subtle changes in ligament integrity and spinal cord compression. While CT remains the gold standard for identifying fractures and osseous abnormalities, MRI’s ability to provide comprehensive visualization of neural structures ensures it remains a critical tool in surgical planning and postoperative assessment [37].

Managing vertebral fractures involves pain control, rehabilitation, and prevention strategies to avoid additional fractures. Acute pain from vertebral fractures may persist for 12 weeks or more, while chronic pain can result from vertebral deformity, paraspinal muscle spasms, degenerative arthritis near the fracture site, or changes in spinal alignment [38]. Exercise programs tailored for elderly individuals with post-vertebral fractures have been shown to reduce analgesic use, improve quality of life, and increase bone mineral density [39,40]. A meta-analysis demonstrated that such exercise programs could prevent or reverse approximately 1% of annual bone loss in the spine and hip regions among older women [39].

For pain management, therapeutic doses of acetaminophen (2.4 g/day) are often effective, with codeine (30 to 60 mg every 6 h) recommended for breakthrough pain [41]. Short-term randomized trials (ranging from 2 to 16 weeks) have also shown that calcitonin provides a rapid analgesic effect [42,43]. Recommended doses of calcitonin for pain relief are administered subcutaneously or intranasally and are given daily, with 50 to 100 IU administered subcutaneously or 200 IU intranasally [44]. Intercostal nerve blocks may also relieve pain in patients with vertebral fractures [45].

Supportive bracing is commonly recommended for osteoporotic vertebral fracture (OVF) patients. Braces such as three-point contact braces, hyperextension orthoses, Jewett braces, or thoracolumbar sacral orthoses (TLSOs) have shown benefits in terms of trunk muscle strength, posture, quality of life (QOL), activities of daily living (ADLs), and pain relief. A prospective randomized study highlighted the substantial benefits of TLSO use [46]. A systematic review found that spinal orthoses improved functional outcomes in neurologically intact patients aged 60 and older, reducing kyphotic deformity, enhancing postural stability, and increasing muscular strength [47].

Vertebral augmentation (VA) procedures, including kyphoplasty (KP) and vertebroplasty (VP), have been extensively studied. VA offers advantages such as using local anesthesia, mechanical stabilization through cement injection, and analgesic effects from the thermal reaction of polymethyl methacrylate (PMMA) cement [48]. Studies suggest that VA can effectively correct local kyphosis and alleviate pain, significantly improving sagittal alignment [49].

Vertebral augmentation (VA) is the primary surgical intervention for osteoporotic vertebral compression fractures (OVCFs), valued for its minimally invasive approach, reduced blood loss, rapid postoperative recovery, and high safety profile [50]. The most commonly used VA techniques are percutaneous vertebroplasty (PVP) and percutaneous kyphoplasty (PKP).

In PVP, bone cement is injected into the vertebral body under high pressure, making the procedure relatively straightforward. This technique eliminates the need for dilator placement, as repeated punctures to establish a dilator channel are unnecessary, resulting in minimal puncture-related complications [51].

However, PVP does not provide a confined space for bone cement, leading to a reported cement extravasation rate ranging from 11% to 76%. Despite this, strict adherence to surgical protocols and extensive physician experience can significantly mitigate the risk of cement leakage. These measures ensure that PVP remains a cost-effective and reliable surgical option for managing OVCFs [52].

Polymethylmethacrylate (PMMA)-based bone cements play a critical role in stabilizing vertebral compression fractures, with their mechanical performance highly dependent on material composition and the inclusion of specific additives. Recent studies demonstrate that the addition of admixtures like glassy carbon (GC) can significantly enhance PMMA’s compressive strength and hardness, although the effect varies with particle size and concentration. Fine-grained GC particles (0.4–12 µm) maintain mechanical integrity even at higher concentrations, while coarse-grained GC particles (20–50 µm) can disrupt polymerization and reduce strength. Similarly, hydroxyapatite (HA) has been shown to enhance bioactivity and osteointegration, but excessive concentrations above 5% negatively impact key mechanical properties, including tensile strength and elasticity [53,54].

Tricalcium phosphate (TCP) has also emerged as a valuable additive, improving the bioactivity and osteoconductivity of bone cements while promoting bone regeneration. However, excessive concentrations of α-TCP above 3% may compromise mechanical stability, whereas β-TCP provides better compatibility and preserves structural integrity. These findings highlight the importance of optimizing admixture formulations to balance mechanical stability with biological compatibility.

Carefully tailoring PMMA-based bone cements with specific additives, such as GC, HA, or TCP, allows for a personalized approach in vertebral augmentation procedures, ensuring improved clinical outcomes by combining durable mechanical properties with long-term biological benefits. This approach underscores the necessity of selecting the right admixtures to meet the unique demands of each clinical scenario [55].

## 3. Spondylolisthesis

Spondylolisthesis is a spinal disorder characterized by the anterior displacement of one vertebral body over another. Differentiating between isthmic and degenerative spondylolisthesis is essential, as they stem from distinct pathophysiological mechanisms and present with different clinical implications. Isthmic spondylolisthesis arises due to a defect in the pars interarticularis, typically resulting from repetitive mechanical stress. This defect can progress to spondylolysis, ultimately leading to vertebral slippage. This condition is most frequently observed in younger individuals, particularly those engaged in athletic activities that involve lumbar hyperextension [56]. Conversely, degenerative spondylolisthesis occurs in the absence of a pars defect and is primarily driven by age-related degeneration of the intervertebral disks and facet joints, leading to segmental instability. This form predominantly affects the elderly population and is often associated with lumbar spinal stenosis, which can result in neurogenic claudication and progressive functional impairment [15,57]. Given the distinct etiological and clinical profiles of these two subtypes, precise diagnosis is paramount to guiding optimal management strategies, which may include conservative treatment, pain management, or surgical stabilization, depending on severity and symptomatology.

Degenerative spondylolisthesis (DS), a common condition in older adults, is a progressive spinal disorder and a leading cause of lumbar spine surgery [58]. Unlike dysplastic or isthmic types, DS is characterized by forward displacement of a vertebra while maintaining an intact posterior arch, most frequently occurring at L4 on L5. This condition typically arises from disk dehydration, which increases stress on the facet joints and leads to lumbar spinal stenosis (LSS) and spinal deformity [59]. Symptoms vary widely, including back and leg pain, radiculopathy, and neurogenic claudication [60].

While surgical intervention is supported by randomized trials [61], the optimal treatment approach—whether decompression alone or combined with stabilization—remains a topic of debate [62,63,64]. The variability in DS management underscores the lack of standardized, evidence-based guidelines [65,66]. Several classification systems have been developed to assist with diagnosis and treatment planning. Meyerding’s 1932 scale grades vertebral slippage but has limited applicability to DS, as most cases involve mild slippage (<30%) [67]. The French Society for Spine Surgery (FSSS) classification incorporates sagittal alignment to guide treatment decisions [68,69], while the CARDS classification, introduced in 2014, emphasizes disk height and vertebral translation, as well as clinical features such as leg pain [69,70].

In 2015, the Degenerative Spondylolisthesis Instability Classification (DSIC) was introduced, focusing on stability assessment. In 2019, Kobayashi et al. examined sagittal alignment using the sagittal vertical axis (SVA) to study DS progression [71]. More recently, the 2020 Kulkarni scoring system evaluated the necessity of spinal fusion based on various clinical and radiographic factors [72,73]. Although these classification systems offer valuable insights, they often fail to integrate the structural and clinical factors necessary for personalized DS management. This highlights the ongoing need for a more comprehensive classification framework.

The Spinal Deformity Study Group (SDSG) classification system aimed to address the limitations of earlier classification systems by incorporating the Meyerding classification to evaluate the severity of displacement, degree of dysplasia, and sacropelvic balance, aiding in surgical decision-making. The Meyerding classification determines the grade of slip based on the degree of vertebral displacement, which is measured using standing, neutral lateral radiographs of the lumbar spine.

The system categorizes the slip into five grades:

- Grade I: 0% to 25% slip.
- Grade II: 25% to 50% slip.
- Grade III: 50% to 75% slip.
- Grade IV: 75% to 100% slip.
- Grade V: Greater than 100%, also known as spondyloptosis.

Grades I and II are classified as low-grade slips, whereas Grades III, IV, and V are considered high-grade slips. For low-grade slips (Grades I and II), treatment typically involves decompression and/or fusion. Decompression alone is preferred when there is minimal instability, but fusion may be required when iatrogenic instability is caused by decompression.

In contrast, the management of high-grade slips (Grades III, IV, and V) depends on additional factors, including mobility on flexion and extension, sagittal alignment, etiology, and disk height. Surgical approaches for high-grade slips generally involve a combination of fusion and decompression to restore stability and address symptoms [74].

Evaluating a patient with symptomatic lumbar spondylolisthesis (SPL) involves a combination of history-taking, physical examination, and imaging studies to identify both “red flags” (e.g., serious spinal conditions like cauda equina syndrome or infection) and “yellow flags” (e.g., psychosocial factors such as fear avoidance, pain catastrophizing, and low self-efficacy that may impede recovery) [75,76,77]. An accurate diagnosis is essential since SPL symptoms often mimic non-specific lower back pain (LBP). SPL-related pain typically worsens with prolonged positions or certain movements, while discogenic pain intensifies during forward bending and facet joint pain increases with spinal extension [78]. If SPL leads to nerve root compression, symptoms can include paresthesia, sensory loss, or neurogenic claudication, limiting mobility and daily activities [79]. Tools such as pain drawings, the Oswestry Disability Index, and psychological questionnaires (e.g., assessing self-efficacy and coping strategies) are valuable for evaluating pain characteristics and their impact on quality of life, aiding in treatment planning [80,81,82].

Imaging plays a crucial role in diagnosing SPL. Standing lateral X-rays are the standard for detecting degenerative SPL, while flexion–extension X-rays assess instability, defined as a vertebral shift of more than 3 mm or rotation exceeding 10° [83,84]. Advanced MRI techniques and functional imaging further support the evaluation of early instability and help differentiate between stable and unstable SPL, particularly in younger patients [85,86].

Physical examinations for SPL include tests for instability and segmental mobility, such as the step-off sign and Posterior Shear Test (PST), though the reliability of these tests can vary [87]. Provocation tests like the Prone Instability Test (PIT) and Passive Lumbar Extension Test (PLET) are useful for identifying SPL-related pain, with PLET demonstrating high sensitivity and specificity for detecting SPL instability [88,89]. By integrating clinical assessments, imaging, and targeted physical tests, clinicians can effectively distinguish between stable and unstable SPL, facilitating a more individualized and precise approach to managing the condition.

## 4. Surgical Techniques and Instrumentation for Spinal Stabilization

A recent study on spinal stabilization in multiply injured patients concluded that early stabilization of thoracolumbar fractures—particularly within the first 72 h after hospital admission—can bring significant benefits. Specifically, patients who received early spinal surgery demonstrated reduced ventilation time, lower acute respiratory distress syndrome (ARDS) rates, decreased multiple organ failure occurrence, and shorter ICU and hospital stays. The data particularly highlighted that early intervention was advantageous for those with thoracic spine injuries (AIS score ≥ 4), showing a reduction in sepsis rates and other complications related to prolonged immobilization. Based on these findings, the study suggests prioritizing early, minimally invasive posterior stabilization for patients with severe thoracic trauma combined with spine fractures, supporting a proactive surgical approach to optimize patient outcomes [90].

A particular pathology that requires surgical stabilization for improved patient outcomes is ankylosing spondylitis (AS). This chronic condition causes progressive fusion of the spine, known as the “bamboo spine”, which significantly weakens its structural integrity. In patients with AS, even minor trauma can lead to severe spinal fractures due to the spine’s reduced flexibility and underlying osteoporosis [91]. A recent study from the Swedish National Registry demonstrated that surgical stabilization of spinal fractures in AS patients led to notably better survival rates than nonsurgical treatment, even though the surgical approach comes with a risk of complications. Patients undergoing surgery had a lower mortality rate, with spinal cord injuries identified as a critical factor influencing outcomes. The study underscores the importance of prompt and effective stabilization, advocating for surgical intervention to prevent life-threatening complications and improve overall survival in patients with AS-related spinal fractures [92].

Spinal instrumentation is a critical component in the surgical management of spondylolisthesis, particularly for patients who do not respond to conservative treatments and experience severe, persistent symptoms [93,94]. Historically, approaches to degenerative spondylolisthesis involved neural decompression, fixation, and fusion, with techniques evolving as new technologies emerged [95]. The development of transpedicular screws and advancements in spinal instrumentation have enabled effective interbody fusion methods such as transforaminal lumbar interbody fusion (TLIF), which provides stability and promotes fusion-like invasive surgery (MIS) techniques, alongside stereotactic spinal guidance, further enhancing patient outcomes by reducing muscle injury, minimizing perioperative pain, and improving the precision of screw placement. Despite whether to reduce vertebral slippage or proceed with in situ fusion, emerging evidence suggests that partial reduction may benefit patients with high-grade slips by improving alignment and biomechanical stability [96]. Nevertheless, as current studies are often limited in scale, continued research through randomized trials and data from surgical registries are essential to refine surgical strategies and establish standardized guidelines.

The selection of a surgical approach for interbody fusion—whether anterior, posterior, or lateral—depends on various clinical factors, including patient comorbidities, the specific spinal level involved, and the surgical objectives. Anterior approaches, such as ALIF, offer direct access to the intervertebral disk space with minimal disturbance to posterior musculature, leading to shorter operative times and fewer unplanned reoperations. However, these approaches carry a higher risk of complications like deep vein thrombosis (DVT) and pulmonary embolism due to the manipulation of major abdominal vessels. Posterior approaches (PLIF/TLIF) are better suited for direct neural decompression and are often preferred for higher lumbar levels or cases with complex posterior pathology. Lateral approaches, such as XLIF and OLIF, avoid anterior vessel manipulation and provide access to multiple levels, though they can be technically challenging and less feasible at higher lumbar levels. Patient-specific characteristics, including obesity and related comorbidities, significantly influence the choice of approach. For example, while anterior approaches may lower reoperation rates in obese patients, they increase the risk of thromboembolic complications, underscoring the importance of tailoring surgical strategies to the individual patient [97].

The transforaminal lumbar interbody fusion (TLIF) technique, popularized by Harms and Rolinger, provided a unilateral approach to achieving the same level of circumferential fusion as the posterior lumbar interbody fusion (PLIF) technique [98].

Although open TLIF produced excellent results, it was associated with significant morbidity due to iatrogenic soft tissue and muscle injury caused by subperiosteal stripping of the paraspinal muscles and prolonged retractor use. This set of complications, characterized by postoperative back pain, delayed recovery and ambulation, reduced trunk muscle strength, and poorer long-term outcomes, became known as “fusion disease”. To achieve comparable or better outcomes with a smaller surgical footprint, Foley introduced the minimally invasive TLIF (MI-TLIF) approach. By utilizing smaller incisions and accessing the spine through paraspinal muscle-splitting corridors, MI-TLIF significantly reduces soft tissue and muscle disruption [99].

On the other hand, ACDF, often employed for cervical pathologies, allows for direct anterior access to the cervical spine, facilitating the removal of herniated disks and restoration of cervical alignment. According to recent studies, both techniques demonstrate high fusion rates, but the choice between them is influenced by patient-specific factors, including the spinal level involved and the presence of comorbidities [100,101].

Three-dimensional printing (3DP) and minimally invasive spine surgery (MISS) are advancing spinal procedures by enhancing precision and reducing patient recovery times. Three-dimensional printing enables the creation of patient-specific tools and implants, such as pedicle screw guides and interbody cages, which improve accuracy, reduce intraoperative risks, and enhance load distribution. Despite its potential, 3DP adoption is limited by significant time and cost requirements. Long-term complications like adjacent segment disease (ASD) remain a concern, as studies have shown no significant difference in ASD rates between open and minimally invasive approaches over a decade. These advancements highlight the need for individualized surgical planning and long-term follow-up to optimize outcomes [102].

Interbody fusion procedures seek to increase the intervertebral space enough to place a cage, providing stability between vertebrae until full fusion. This expansion helps preserve disk height and spinal curvature by stretching the annulus and supportive ligaments. Most cages feature a hollow center filled with bone grafts or osteobiologics that promote fusion, and some have porous structures that enhance osteoconductive properties [103]. Since fusion typically requires 6–12 months, immediate stability is essential, often achieved with pedicle screws and rods. For anterior or lateral approaches, certain cages incorporate screws or wedges to offer immediate stabilization, eliminating additional positioning steps for screw placement [104]. These cages vary in their construction materials, such as titanium, PEEK, and biologic options, with an ideal design matching the bone’s modulus to prevent subsidence and using materials that support osteoconductivity and radiolucency to facilitate fusion assessment [105].

Titanium cages are favored for their strength and high osteoconductivity, though their rigidity compared to bone can lead to endplate damage and subsidence, along with potential imaging interference [106]. With bone-matching elasticity, PEEK cages reduce subsidence and provide radiolucency for clearer imaging, though their hydrophobic surface may limit bone integration [107]. Titanium cages offer superior osseointegration and structural stability, which significantly lower the risk of subsidence and revision surgeries, particularly in the lumbar spine. In contrast, PEEK (polyetheretherketone) cages, with an elasticity modulus closer to that of cortical bone, help minimize stress shielding and allow better imaging due to their radiolucency. However, their hydrophobic surface limits protein absorption and cell adhesion, potentially compromising osseointegration. A recent meta-analysis revealed that while titanium cages have a lower rate of subsidence and revision surgeries compared to PEEK in the lumbar spine, these differences are not statistically significant in cervical spine applications. These findings suggest that material selection should consider spine region and individual patient factors to optimize clinical outcomes [108]. Hybrid cages, combining PEEK bodies with titanium edges, aim to capitalize on the strengths of both materials, minimizing stress shielding and subsidence while supporting fusion. Biologic implants, such as allogenic bone grafts, offer a modulus close to the bone with radiolucency but may risk fracturing upon insertion [108,109,110].

Surgical treatment for spinal fractures is crucial in cases of mechanical instability, neurologic injury, severe kyphosis over 30 degrees, or when nonoperative methods fail, leading to deformity or worsening symptoms. Surgical approaches—anterior, posterior, or combined—are tailored to fracture type and surgeon preference, using fixation methods like pedicle screws for thoracic/lumbar fractures and lateral mass screws for cervical fractures. Both open and minimally invasive techniques, including percutaneous screw fixation, enhance precision with tools like fluoroscopy, navigation, and emerging robotic assistance. In elderly patients with osteoporotic fractures, procedures like vertebroplasty and kyphoplasty improve stability and pain relief. Special considerations are needed for conditions like ankylosing spondylitis and DISH, where unstable fractures pose high neurologic risks, often benefiting from cement augmentation for added support [111].

## 5. Postoperative Outcomes and Complications

Informed consent is a cornerstone of patient-centered care, requiring a comprehensive discussion of the risks, benefits, and alternatives to surgical treatment. This process should ensure patients can make decisions aligned with their values and preferences, emphasizing clear communication. Variability in risk perception between patients and surgeons can significantly influence decision-making, underscoring the need for tailored conversations. It has been highlighted that healthcare professionals have a professional and ethical obligation to facilitate these discussions, as mandated by legal frameworks in multiple jurisdictions. Effective informed consent fulfills legal requirements and strengthens the therapeutic alliance by fostering trust and shared decision-making, thus mitigating potential disputes and promoting patient autonomy.

Furthermore, variability in how risks and outcomes are perceived highlights the need for surgeons to adopt a patient-specific approach during consultations. By prioritizing open dialog and adjusting explanations to the patient’s level of understanding, healthcare professionals can bridge gaps in perception and foster better-informed decisions [112].

A recent study highlights significant variability in the effectiveness of spinal surgeries, with many patients failing to achieve meaningful quality-of-life improvements. Poor pain control within the first 24 h post-surgery was identified as a key predictor of worse outcomes on the Oswestry Disability Index (ODI) and Neck Disability Index (NDI) up to two years later. The CAPPS score, validated in the study, predicts poor pain control and long-term outcomes, with each score increasing the likelihood of failing to achieve clinically meaningful improvement by 10%. Addressing modifiable preoperative risk factors, such as opioid use, severe pain, and depression, is critical. The study also found that most clinical improvements occur within the first year post-surgery, with about 40% of patients failing to reach the MCID threshold. These findings align with prior research and emphasize improving outcomes by integrating Enhanced Recovery After Surgery (ERAS) principles—such as preemptive analgesia, minimally invasive techniques, and multimodal pain management. Incorporating the CAPPS score into preoperative planning can help identify high-risk patients and guide tailored interventions, underscoring the critical role of pain management in achieving surgical success [113].

Regarding spinal fracture treatment, a recent meta-analysis compared surgical approaches, including traditional open, Wiltse, and percutaneous techniques, focusing on postoperative outcomes and complications. Minimally invasive approaches, particularly the Wiltse and percutaneous methods, demonstrated significant advantages over the open approach, such as reduced operative times, lower perioperative blood loss, and shorter hospital stays. For instance, the percutaneous approach significantly improved postoperative Oswestry Disability Index (ODI) scores and lower visual analog scale (VAS) pain scores than the open approach, indicating better pain management and functional outcomes. However, differences in Cobb angle, vertebral body angle, and vertebral height were minimal among the approaches, suggesting comparable structural results. Despite these benefits, minimally invasive techniques require greater surgical expertise, as complications like screw malpositioning and extended fluoroscopy times have been reported, particularly during the learning curve. These findings emphasize the evolving role of less invasive techniques in improving patient recovery while minimizing complications [111].

Traumatic spinal fractures treated with osteosynthesis, even in the absence of initial neurological deficits, pose significant long-term challenges for patients. Over a 12-year follow-up period, many individuals reported persistent back pain that severely affected daily, professional, and social aspects of their lives, as evidenced by elevated Dallas questionnaire scores. This chronic pain often required ongoing analgesic use, underscoring its organic nature rather than psychological origins, given the relatively lower impact on anxiety levels.

Professionally, the burden was considerable, with more than half of the patients requiring extended sick leave exceeding six months, though 60% were eventually able to resume their previous work. Interestingly, leisure and sports activities were not markedly altered compared to controls, yet the overall picture of chronic pain and its repercussions on personal and familial domains reflects the difficulty of achieving full recovery. These findings highlight the importance of addressing the mechanical stabilization of spinal fractures and the broader, long-term implications for patient well-being and functionality [114].

Postoperative outcomes in spinal fusion surgery for degenerative lumbar spondylolisthesis (DLS) vary significantly among patients, with approximately 28% experiencing persistent lower back pain (LBP) two years after surgery. Predictive factors for these outcomes include age, preoperative lumbar spinal stenosis severity, and paraspinal muscle composition, particularly fatty infiltration and the functional cross-sectional area of the erector spinae. Advanced machine learning models, such as XGBoost version 1.3, demonstrated superior accuracy in identifying high-risk patients by integrating these variables, achieving an area under the curve (AUC) of 0.81. These findings highlight the importance of addressing modifiable preoperative conditions and utilizing predictive analytics to enhance surgical planning and patient counseling, ultimately improving long-term recovery trajectories [115].

## 6. Conclusions

Spinal stabilization remains a cornerstone in managing spinal fractures and spondylolisthesis, addressing structural instability, alleviating pain, and improving patient function. This review highlights the diverse types and causes of spinal fractures, from traumatic events to degenerative conditions like osteoporosis, alongside a thorough exploration of diagnostic tools and classification systems such as the AO Spine classification. Similarly, understanding its types, grading, and pathophysiology for spondylolisthesis is critical to tailoring management strategies that consider biomechanical changes and patient-specific risk factors.

Surgical interventions for spinal stabilization, particularly spinal fusion, and decompression techniques, have evolved with advancements in instrumentation, including pedicle screws, rods, interbody cages, and minimally invasive approaches augmented by navigation and robotic systems. These innovations aim to improve precision, reduce surgical morbidity, and enhance patient recovery. However, postoperative outcomes remain variable, with metrics like pain relief, disability scores, and fusion rates highlighting both successes and challenges, such as hardware failure and adjacent segment disease.

## Figures and Tables

**Table 1:** AO Spine classification system.

Type	Description
Type A	Simple fractures with minimal fragmentation.
Type A1	Minimal displacement, indicating low instability.
Type A2	Greater displacement than Type A1, indicating moderate instability.
Type A3	Extensive displacement, making them the most unstable among Type A fractures.
Type B	Partial articular fractures involve the joint surface but do not extend through it completely.
Type B1	Involves a single articular surface with low instability.
Type B2	Affects two articular surfaces, exhibiting moderate instability.
Type B3	Involves multiple articular surfaces with high instability.
Type C	Complete articular fractures extending through the joint surface, highly unstable.
Type C1	Extra-articular fractures occur away from the joint.
Type C2	Involve the metaphyseal region and extend into the joint.
Type C3	Extend into the joint, affecting both the metaphysis and diaphysis.

**Table 2:** TLICS classification system.

Severity Score	Description
Score 1–3	Nonoperative treatment is recommended.
Score 4	Operative or nonoperative treatment based on neurological status and injury characteristics.
Score ≥ 5	Operative treatment is recommended.
